# Having a family doctor was associated with lower utilization of hospital-based health services

**DOI:** 10.1186/s12913-015-0705-7

**Published:** 2015-01-28

**Authors:** Colman SC Fung, Carlos KH Wong, Daniel YT Fong, Albert Lee, Cindy LK Lam

**Affiliations:** Department of Family Medicine and Primary Care, the University of Hong Kong, 3/F., 161 Main Street, Ap Lei Chau Clinic, Ap Lei Chau, Hong Kong; School of Nursing, the University of Hong Kong, 4/F, William M.W. Mong Block, 21 Sassoon Road, Pokfulam, Hong Kong; Centre for Health Education and Health Promotion, The Jockey Club School of Public Health and Primary Care, the Chinese University of Hong Kong, 4/F, Lek Yuen Health Centre, 9 Lek Yuen Street, Shatin, Hong Kong

**Keywords:** Family doctor, Health utilization, Hospitalization, Emergency services, Chinese, Count data

## Abstract

**Background:**

Primary care in the United States and most countries in Asia are provided by a variety of doctors. However, effectiveness of such diversified primary care in gate-keeping secondary medical services is unknown. This study aimed to evaluate health services utilization rates of hospital emergency and admission services among people who used different primary care doctors in Hong Kong.

**Method:**

This study was a population-based cross-sectional telephone survey using structured questionnaire on health services utilization rates and pattern in Hong Kong in 2007 to 2008. Information on the choice of primary care doctors, utilization rates and patterns of primary care service were collected. Poisson and logistic regression analyses were used to explore any differences in service utilization rates and patterns among people using different types of primary care doctors.

**Results:**

Out of 3148 subjects who completed the survey, 1896 (60.2%) had regular primary care doctors, of whom 1150 (60.7%) regarded their regular doctors as their family doctors (RFD). 1157 (36.8%) of them did not use any regular doctors (NRD). Only 4.3% of the RFD group (vs 7.8% of other regular doctors (ORD) and 9.6% of NRD) visited emergency service and only 1.7% (vs 3.6% of ORD and 4.0% of NRD) were admitted to hospital for their last episode of illness. Regression analyses controlling for sociodemographics and health status confirmed that respondents having RFD were less likely to use emergency service than people who had NRD (OR 0.479) or ORD (OR 0.624) or being admitted to hospital (OR 0.458 vs NRD and 0.514 vs ORD) for their last episode of illness.

**Conclusion:**

Primary care is the most effective in gate-keeping secondary care among people with regular family doctors. People without any regular primary care doctor were more likely to use emergency service as primary care. The findings supported a family doctor-led primary care model.

**Trial registration number:**

ClinicalTrials.gov ID: NCT01422031.

**Electronic supplementary material:**

The online version of this article (doi:10.1186/s12913-015-0705-7) contains supplementary material, which is available to authorized users.

## Background

Primary care has a great impact on the population’s health and its effectiveness and accessibility are the key to health for all [1,2]. Primary care should be able to gate-keep hospital health services, [3-10] including accident and emergency service [11]. Experience from western countries where primary care is provided mainly by family doctors or general practitioners with standardized postgraduate training is encouraging [5,8,12-15]. Starfield et al. found that countries with a more uniform type of primary care providers had better health outcomes [5,8]. Primary care provided by family doctors has been found to be the most cost-effective health care service [8-10,12,16,17]. A higher supply of general practitioners and family physicians, but not other primary care doctors, was associated with lower mortality rates [13,14] and higher early cancer detection rates [15,18]. The family-doctor model has been proposed to be the solution for the rising demand for quality primary care services for aging populations in Hong Kong [19] and the United States [20] .

Like the United States, primary care in many countries in Asia is provided by a variety of Western and Chinese medicine practitioners. Instead of seeing a primary care doctor in the community, some people in Hong Kong use emergency service of the hospital as the main provider of primary care for various reasons [21]. However, research found that 57.0% of patients attending emergency service could actually be managed by family doctors [21]. Other studies also found that the population in Hong Kong were twice as likely to consult a doctor when they were ill, compared to patients in the US and UK [22,23]. Consultations with more than one doctor for the same episode of illness (doctor-shopping) was another prevalent phenomenon [24]. So far, no known research has been done to investigate the effectiveness in safeguarding the utilization rates of other health care service by this kind of pluralistic primary care service, and how having a family doctor makes any difference.

This study aimed to evaluate health services utilization rates in particular those of hospital emergency and secondary specialist services among people who used different primary care doctors in Hong Kong.

## Method

Analysis was done on data collected in a cross-sectional general population health service utilization survey that was carried out in two phases in summer (September to October 2007) and winter (March to April 2008). Telephone-owning households in Hong Kong were contacted by random digital dialling by the Social Sciences Research Centre of the University of Hong Kong using the computer-assisted telephone interviewing system with a 95% household coverage rate. Respondent in a selected household was randomly identified by the use of the last birthday rule. The main carer of a minor under age of 18 answered the survey as the proxy. The exclusion criteria were non-residential numbers; Inability to communicate in Cantonese, Putonghua or English; Refusal to telephone interview; and no contact after 5 attempts. Details of the study design are described in previous papers [25-27].

A structured questionnaire concerning the presence and type of regular primary care doctors, prevalence (likelihood) of utilization of different health services for their last illness episode, medical health services utilization rates in the past four weeks, health status and socio-demographics was administered. No time limit was set when asking the respondents’ last illness episode (likelihood), and four weeks was used as the consistent time reference for the recall of all types of health service utilization, including emergency services, hospital admissions, and primary care doctor or family doctor consultations for the monthly health services utilization rates. A total of 5174 eligible households were contacted and 3148 (60.8%) subjects, with 1616 and 1532 in the summer and winter phases respectively, completed the cross-sectional survey.

Subjects were classified into three groups: the first one “Regular Family Doctor group” (RFD) included subjects who reported to have a regular primary care doctor and considered him/her as the family doctor; the second group was “Other Regular Doctor group” (ORD), involving subjects who had a regular primary care doctor whom was not considered as his/her family doctor; the third one was “No Regular Doctor group” (NRD) for subjects had no regular primary care doctor. A regular primary care doctor was defined as the doctor whom the persons who usually consulted when he/she was ill. A family doctor was defined as the doctor whom the person would consult for all their health problems.

The difference in hospital-based health service utilization was investigated in two ways. The first one was by the prevalence (likelihood) of use of the health services during the last episode of illness, while the second one was the monthly utilization rate of hospital-based (emergency service and in-patient) services. It was estimated that 10% of the population belonged to RFD group who would have 0.48 consultations per month [22,28,29]. A minimum sample size of 3000 subjects was estimated in order to be able to detect a 15% difference in number of consultations over a period of 4 weeks across three comparison groups with 80% power and 5% level of significance by Poisson regression.

### Outcome measures

Primary outcome measures were the prevalence (likelihood) of the health services utilization (i.e. utilization of hospital emergency service or admission or consultation with primary care doctors per subject) during the last episode of illness. Secondary outcome measures were the monthly rates of doctor consultation (number of visits to the doctors over the past 4 weeks per subject).

### Data analysis

Multiple logistic regression analyses were used to determine the effects of having a RFD on likelihood of hospital admission, emergency visits or specialist consultations in the last episode of illness by adjusting for possible confounding factors of socio-demographics, health status, lifestyles, chronic morbidity and seasonality. Model goodness-of-fit of logistic regressions was assessed using the Hosmer-Lemeshow test. Utilization rates were compared across three groups by Poisson regression analyses when adjusted for confounding variables. Negative binomial regression models were used instead of Poisson regression models in cases when the ratio of residual deviance to degrees of freedom was far greater than one, indicating the overdispersed count outcomes.

All estimates were accompanied with a 95% confidence interval and a p-value < 0.05 was considered as statistically significant. The SPSS 20.0 Window (SPSS Inc., Chicago, USA) was used to perform the statistical analysis.

This study was approved by the Institutional Review Board of the University of Hong Kong/ Hospital Authority Hong Kong West Cluster (HKU/HA HKW IRB # UW 07–021). The trial registration number for this study is ClinicalTrials.gov ID: NCT01422031.

## Results

Amongst the 3148 subjects, 1896 (60.2%) reported that they had a regular primary care doctor. Of which, 60.7% (1150, 36.5% of total population) claimed their regular doctors as family doctors, while 39.3% (746, 23.7% of total population) claimed their regular doctors not their family doctors. On the whole, 1256 (39.9%) said they had a family doctor and majority (91.6%) of them (1150, 36.5% of total population) said their family doctors were their regular primary care doctors. The remaining 106 subjects (8.4%) who said they had a family doctor but their family doctors were not their regular primary care doctors were excluded from analysis for our study purpose because these subjects did not fit into any of our pre-set three subject groups. Table 1 shows the socio-demographic and morbidity characteristics of the subjects. The RFD group had more subjects who were female (61.9%), had household monthly income > HKD$20,000 (58.0%), and were younger (mean age 38.5 years), when compared with the ORD and NRD groups.

**Table 1:** **Characteristics of subjects by primary care doctor choice groups**

	Total*	**RFD**	**ORD**	**NRD**
	**(n = 3148)**	**(n = 1150)**	**(n = 746)**	**(n = 1157)**
Age (mean ± s.d.) †	40.2 ± 18.1	38.5 ± 17.6	40.1 ± 18.0	41.3 ± 18.5
<25 yrs (%)	23.0	21.9	23.8	24.1
25-64 yrs (%)	68.0	72.8	67.4	63.9
≥65 yrs (%)	9.0	5.3	8.7	12.0
Male (%)	40.9	38.1	39.4	44.9
Married (%)	54.9	57.1	53.6	53.2
Education ≤ primary school (%)	17.4	13.4	17.1	20.7
Household income < HKD$20,000 (%) †‡	53.1	42.0	52.0	64.1
Occupation (%)				
Managerial/ Admin/ Professionals/ Employer	12.5	15.9	12.3	9.7
White collar workers	16.5	19.5	17.5	13.0
Blue collar/ Service sales worker	20.4	17.4	21.7	23.0
Chronic disease (%)	34.7	34.8	38.2	30.8
Long term medications (%)	22.8	23.7	25.1	19.5
General Health condition (%)				
Excellent/Very good/Good	48.7	53.2	41.3	50.0
Fair	44.9	42.3	49.2	44.3
Poor	6.4	4.3	9.5	5.8

Notes: RFD = Regular family doctor group; ORD = Other regular doctor group; NRD = No regular doctor group.

*The sum of three groups did not add up to total, as 95 respondents were not sure if they had regular/ family doctors.

†Significant difference between RFD and NRD by one way ANOVA test or chi-square test, as appropriate.

‡Median monthly domestic household income of Hong Kong population = HKD$17,250 (Census and Statistics Department, 2006).

Multiple logistic regressions found that younger age, currently married, white-collar work, higher household monthly incomes, having a chronic disease, need long-term medications and regular exercise were independent factors associated with having a regular primary care doctor (Table 2).

**Table 2:** **Factors associated with a regular primary care doctor by logistic regression**

**Independent variables**	**Odds ratio**	**95% CI**	**P-value**
Age	0.987	( 0.978 - 0.997 )	0.010
Sex (male)	1.204	( 0.974 - 1.488 )	0.087
Marital status (single/ divorced/ widower)			0.044
– Married	1.333	( 1.047 - 1.697 )	0.019
– Refuse to answer	0.741	( 0.239 - 2.300 )	0.604
Educational level (nil/ primary)			0.092
– Secondary	1.319	( 1.008 - 1.725 )	0.043
– Tertiary	1.082	( 0.774 - 1.512 )	0.645
– Refuse to answer	0.765	( 0.245 - 2.391 )	0.645
Household monthly income (< $5,000)			<0.001
– $5,000 - $9,999	0.868	( 0.551 - 1.369 )	0.543
– $10,000 - $19,999	1.487	( 0.970 - 2.279 )	0.069
– $20,000 - $29,999	2.243	( 1.422 - 3.537 )	<0.001
– $30,000 - $39,999	2.594	( 1.583 - 4.252 )	<0.001
– ≥ $40,000	3.384	( 2.108 - 5.432 )	<0.001
– Refuse to answer	2.056	( 1.311 - 3.224 )	0.002
Occupation (blue-collar worker/ service and sales worker)			0.010
– Managerial/ administrative/professional/ employer	1.362	( 0.953 - 1.946 )	0.090
– White-collar worker	1.525	( 1.116 - 2.084 )	0.008
– Student	1.057	( 0.713 - 1.568 )	0.781
– Home-maker	1.142	( 0.825 - 1.581 )	0.423
– Retired/ unemployed	0.746	( 0.518 - 1.074 )	0.115
– Others	0.961	( 0.446 - 2.074 )	0.920
Chronic disease (yes)	0.738	( 0.573 - 0.950 )	0.018
Long term medication (yes)	0.559	( 0.415 - 0.753 )	<0.001
General Health Condition (poor)			0.467
– Excellent	1.333	( 0.727 - 2.444 )	0.353
– Very good	1.572	( 0.966 - 2.557 )	0.069
– Good	1.381	( 0.865 - 2.206 )	0.177
– Fair	1.358	( 0.874 - 2.110 )	0.173
Smoking (yes)	1.206	( 0.938 - 1.551 )	0.144
Drinking (yes)	1.139	( 0.932 - 1.393 )	0.203
Regular exercise (no/ don’t know)	1.372	( 1.131 - 1.665 )	0.001

Notes: Variable in brackets is the reference category for independent variables.

Logistic regression (enter): Odds ratio <1 (less likely than the reference category), Odds ratio >1 (more likely than the reference category).

### Prevalence (Likelihood) of health services utilization

The prevalence (likelihood) of health services utilization during the last episode of illness of the population is shown in Table 3. Amongst the 3148 subjects, 71.7% consulted a doctor, 7.3% visited emergency service and 3.1% were hospitalized. The RFD group were the least likely to visit emergency service or admitted to the hospital, while there was no significant difference between ORD and NRD groups. The NRD group was significantly less likely to consult but more likely than the others to visit emergency service (9.6%).

**Table 3:** **Prevalence (Likelihood) of the use of Health services during the last episode of illness by primary care doctor choice groups**

**Last episode of illness**	**Total**	**RFD**	**ORD**	**NRD**
	**(n = 3148)**	**(n = 1150)**	**(n = 746)**	**(n = 1157)**
**Had consulted any doctors** (%)*†‡	71.7	80.2	74.7	60.8
▪ Had used any Western doctor (%)*†‡	65.4	77.6	68.1	51.3
-- Consulted family doctor (%)*†‡	30.2	67.0	10.6	7.0
-- Consulted regular PC doctor who is not a family doctor (%)†‡	27.5	16.8	54.7	19.4
-- Consulted other doctors (%)*†	19.7	14.3	13.1	29.0
▪ Consulted Chinese medicine practitioner (%)	12.1	13.1	13.1	10.6
▪ **Visited emergency service** (%)*‡	7.3	4.3	7.8	9.6
▪ **Admitted to the hospital** (%)*‡	3.1	1.7	3.6	4.0

Notes: RFD = Regular family doctor group; ORD = Other regular doctor group; NRD = No regular doctor group.

*Significant difference (p < 0.05) between RFD and NRD by univariable logistic regression.

†Significant difference (p < 0.05) between ORD and NRD by univariable logistic regression.

‡Significant difference (p < 0.05) between RFD and ORD by univariable logistic regression.

### Health service utilization rate

The monthly health service utilization rates based on the number of consultations during the past four weeks reported by study subjects are shown in Table 4. One third of the population had used one or more medical services including 24.7% who had consulted community-based Western medicine doctors, 9.6% consulted Chinese medicine practitioners, 3.7 % had visited emergency service, and 1.2% was admitted to the hospital. The mean primary care consultation rate was 0.71 (95%C.I. 0.66, 0.75) per person with 8% of which provided by the emergency service. The RFD group had the lowest monthly illness rate (0.51), and they had the lowest emergency service utilization rate (0.05). The consultation rate of the NRD group was lower (0.49) than those of the others (0.85) but they were more likely than the RFD group to have used emergency service. The highest utilization rate of emergency service was found in the ORD group.

**Table 4:** **Monthly consultation and service utilization rates by primary care doctor choice groups**

	**Total**	**RFD**	**ORD**	**NRD**
	**(n = 3148)**	**(n = 1150)**	**(n = 746)**	**(n = 1157)**
	**mean ± s.d, (% of subjects)**			
Monthly consultation rate*†	0.71 ± 1.53	0.85 ± 1.73	0.85 ± 1.60	0.49 ± 1.26
	(33.6%)	(39.1%)	(38.3%)	(25.2%)
- Western medicine doctors*†‡	0.43 ± 1.00	0.61 ± 1.22	0.50 ± 1.06	0.20 ± 0.62
	(24.7%)	(33.6%)	(28.5%)	(13.5%)
- Western medicine family doctors*†‡	0.21 ± 0.75	0.50 ± 1.10	0.06 ± 0.41	0.03 ± 0.26
	(12.8%)	(30.1%)	(3.5%)	(2.0%)
- Western medicine regular but not family doctors*†‡	0.22 ± 0.65	0.11 ± 0.40	0.44 ± 0.98	0.17 ± 0.56
	(14.4%)	(8.9%)	(26.1%)	(11.9%)
- Chinese medicine practitioner*†‡	0.24 ± 1.03	0.24 ± 1.04	0.32 ± 1.12	0.19 ± 0.97
	(9.6%)	(10.0%)	(12.5%)	(7.5%)
- Hospital emergency service†‡	0.06 ± 0.45	0.05 ± 0.56	0.08 ± 0.47	0.05 ± 0.29
	(3.7%)	(2.3%)	(5.2%)	(3.8%)
**Monthly Hospital admission rate**	0.01 ± 0.14	0.01 ± 0.14	0.02 ± 0.15	0.01 ± 0.13
	(1.2%)	(1.0%)	(1.3%)	(1.1%)

Notes: RFD = Regular family doctor group; ORD = Other regular doctor group; NRD = No regular doctor group.

*Significant difference between RFD and NRD by univariable Poisson regression.

†Significant difference between ORD and NRD by univariable Poisson regression.

‡Significant difference between RFD and ORD by univariable Poisson regression.

### Effects of primary care doctor choice on health service utilization

The differences in outcomes between different primary care doctor choice groups were compared pair-wise by multiple regressions on the data, adjusting for all possible confounders.

Table 5 shows the results of multiple logistic regressions of doctor choice groups on the pattern of service utilization during the last episode of illness. RFD group was more likely than the NRD and ORD groups to have consulted. RFD had around 38-54% lower odds of using emergency service or hospital admissions than the ORD and NRD groups, but there was no difference between the ORD and NRD groups.

**Table 5:** **Effects of primary care doctor choice on use of health services during the last episode of illness**

**Logistic regression results**		**RFD vs. NRD†**	**RFD vs. ORD†**	**ORD vs. NRD†**	**Other significant independent variables (p < 0.05)‡**
	**Overall significance**	**Odds ratio (95% C.I.)**			
Any primary care doctor consultation	0.012	*2.486	*1.342	*1.853	Drinking, regular exercise, occupation, marital status
		(2.053,3.010)	(1.074,1.676)	(1.509,2.275)	
Emergency service visit	<0.001	*0.479	*0.624	0.768	Age, general health, long-term medication, household monthly income, district
		(0.330,0.695)	(0.411,0.949)	(0.536,1.098)	
Hospital admission	0.032	*0.458	*0.514	0.891	Age, long-term medication
		(0.267,0.788)	(0.284,0.932)	(0.540,1.470)	

Notes: RFD = Regular family doctor group; ORD = Other regular doctor group; NRD = No regular doctor group.

*Statistically significant (p < 0.05) difference.

†as reference category for doctor choice groups in logistic regression, Odds ratio <1 (less likely than the reference category), Odds ratio >1 (more likely than the reference category).

‡Adjustment of confounding factors including socio-demographics, health status, chronic morbidity and lifestyle (Additional file 1).

Negative Binomial regressions were used for overdispersed count outcomes of monthly consultation rate whereas monthly emergency service visit rate and monthly hospital admission rate were tested by Poisson regressions (Table 6). Seasonality (summer vs. winter) was also adjusted for and treated as a covariate. The RFD and ORD groups had 54.0% to 64.7% more consultations than the NRD group, with no difference between the RFD and ORD groups. After controlling for confounding variables, the difference in emergency service or hospital admission rates between doctor choice groups was not statistically significant. Seasonality had the strongest effect on service utilization rates, with higher rates found in the winter.

**Table 6:** **Effects of primary care doctor choice on monthly consultation and health service utilization rates**

		**RFD vs NRD†**	**RFD vs ORD†**	**ORD vs NRD†**	**Other significant independent variables (p < 0.05)‡**
	**Overall significance**	**Coefficient (95% C.I.)**			
*Negative Binomial Regression Results*					
Monthly consultation rate	<0.001	*0.473	0.046	*0.427	Seasonality, sex, general health, chronic disease, drinking, occupation, educational level, district
		(0.332,0.614)	(−0.098,0.191)	(0.273,0.581)	
*Poisson Regression Results*					
Monthly emergency service visit rate	0.158	0.253	−0.099	0.352	Seasonality, sex, age, general health, long-term medication, occupation, household monthly income, educational level, district
		(−0.140,0.647)	(−0.490,0.292)	(−0.038,0.742)	
Monthly hospital admission rate	0.360	−0.047	−0.253	0.206	Seasonality
		(−0.764,0.670)	(−1.013,0.507)	(−0.553,0.965)	

Notes: RFD = Regular family doctor group; ORD = Other regular doctor group; NRD = No regular doctor group.

*Statistically significant (p < 0.05) difference.

†as reference category in Poisson/Negative Binomial regression , +ve: positive relationship, −ve: negative relationship.

‡Seasonality with phase I data coded as Summer and phase 2 data coded as Winter was entered as a covariate, and adjustment for confounding factors including socio-demographics, health status, chronic morbidity and lifestyle (Additional file 1).

## Discussion

This study found that the public prone to differentiate family doctors from other primary care doctors, based on the function of the doctor. Around two-third (67.0%) of people having regular family doctor consulted their own family doctors for their last episode of illness. We defined a regular primary care doctor as a doctor whom one would first consult when one needs to, and a family doctor as a doctor whom one would consult for all types of health problems. About two-third (60.2%) of the subjects said that they had a regular primary care doctor, which was similar to the 56% found in an earlier study [28]. More than one-third (36.5%) said that they had a family doctor as their regular primary care doctor; which was higher than the 10% reported by the earlier study that used a narrower definition based on qualification for family doctor [28]. People who were younger, employed as white-collar workers and having higher incomes were more likely to have a family doctor, probably because they were more health conscious as indicated by a higher likelihood of regular exercise. A higher awareness and a better access to family-doctor led primary care may also be the reasons for the difference.

### Prevalence (Likelihood) of health service utilization and utilization rates

An important function of primary care is to gate-keep hospital emergency and admission services. Consistent with local and oversea findings, only a very small minority (1.2% in 4 weeks) of people needed in-hospital treatment and 90% of the medical service was provided by primary care excluding the emergency service (Table 4). People with RFD were about 50% less likely than others to have visited the emergency service or being hospitalized for their last episode of illnesses, after controlling for confounding factors including health status (Table 5). It should be noted that having other regular primary care doctors did not significantly reduce the use of emergency service or hospital services, when compared with people without any regular primary care doctor. Our findings are coherent with that patient-centred care, which was a key element of family medicine, was related to a significantly decreased annual number of visits for specialty care and less frequent hospitalizations [30].

About one third of the population had used primary medical services (33.6%). The primary care service utilization rate (33.5%) was similar to the 37.2% of primary care consultations found in the 2002 Thematic Household Survey, although the prevalence of emergency service visit and Chinese medicine practitioner consultations reported by our subjects were higher [22]. About 67% of RFD subjects consulted their family doctors during the last episode of illness and 54.7% of the ORD group consulted their regular doctors, validating their claims of having a regular primary care doctor and showing that there was a better continuity of care with a family doctor.

Availability and accessibility of primary health care influenced the rate of hospitalization in which an accessible and readily available primary health care system lowered the hospitalization and admission rate [31,32]. Continuity of care, which was a key feature of the practice of family medicine, was shown to be associated with a decreased future likelihood of hospitalization [33], and it was also demonstrated in our study that having a regular family doctor reduced their likelihood of using emergency service or secondary hospital services. In other words, people with regular family doctors had easier access to primary care and thus led to a lower demand for hospital-based (both emergency and in-hospital) health services. On the other hand, our study showed that people with RFD consulted doctors more frequently in the past 4 weeks amongst the three groups of people, and their hospital admission rate was the lowest amongst the three groups. A study found out that the visit rates of general practitioners correlated negatively with the rates of hospitalizations [34] and an engagement with a good and effective primary care program reduced the use of emergency services [35,36]which to some extent compatible with the lowest odd ratio for the RFD group for the emergency service usage and hospital admission.

The population-adjusted mean monthly consultation rate was 0.7 (95% CI 0.65, 0.75), which is equivalent to 8.4 (95% CI. 7.8, 9) consultations per year [25]. The rate was consistent with the 9 consultations per year found in the population survey in 1998 [23], and the monthly Western medicine consultation rate was similar to the 0.48 found in another local population survey in 1998 [29], supporting the consistency of the results. There was an impression that the population in Hong Kong used more health services than overseas populations [22,23]. The annual consultation rate of the Hong Kong population was actually similar to the 6.8 and 8.7 found in the US young adult men and women, respectively [30]. Although the figure was much higher than that found in Canada (median 3.3) [31], it was still much lower than the 13.4 visits in Taiwan [32]. The projected annual western medicine consultation rate was around 5 that is in par with that found in Australia and the United Kingdom [33,34]. It is interesting to note that the Chinese medicine practitioner consultations by the 9.5% subjects made up one third (33.5%) of the total volume of medical service used. The ‘excess’ in consultation rates found in the Hong Kong population was the result from the concomitant use of Western and Chinese medicine consultations, which are often not counted in studies in Western countries. This implied that users of Chinese medicine were more likely to have repeated consultations. There is no standard on the optimal consultation rate for a population, which is dependent on the complex interaction between patients’ perceived needs, availability and accessibility of doctors and payment methods [30,31,35].

It seemed contradicting to find that the RFD group had the highest consultation rate when they reported the lowest illness rate. The strongest determinants of health service utilization are perceived health need and accessibility of services [37]. Having a family doctor or other regular primary care doctor probably does not alter a person’s perception of illness but increases accessibility to care. People with regular primary care doctors may also be more inclined to consult because they had positive experience from previous consultations. The possibility of lower ability of coping with illness and higher doctor dependence among people with RFD deserve further research. The findings alerted to the problem of low accessibility to community-based primary care doctor service in the NRD group who may then rely more on emergency service as a source for primary care.

### Limitations

A loose definition of the family doctor was used in this study and the classification into RFD, ORD, and NRD groups was based on subjective self-reporting and its accuracy cannot be verified. This might have affected the results on the between-group differences, but the bias would be more likely to under-estimate rather than over-estimate the difference. It was interesting to note that a small percentage of ORD and NRD groups (people who stated that they did not have a family doctor as their regular primary care doctor) reported visiting their family doctors in their last episode of illness. This reflected that some people may have their own interpretation of what a family doctor should be. Indeed the definition of a family doctor from the patients’ perspectives deserves further exploratory studies.

The study sample was not fully representative of the general population with an over representation of people who were female, younger, and better educated. We tried to adjust for these confounding variables in the regression analyses so that the results can be more generalizable. The results of our analyses were mainly based on the data from the cross-sectional study, which is subject to recall bias and the uncertainty of a causal or effect relationship. Also, the emergency service visit and hospital admission rate were based on 4-week period instead of longer period (e.g. one-year), which may contribute to the low monthly rate obtained. This might have underestimated the rates of use of these health services.

## Conclusions

Primary care is the most effective among people with a regular family doctor in terms of gate-keeping of emergency and hospital in-patient services in the Hong Kong health care system with a variety of primary care doctors. The concepts of primary care and family doctor are being recognized by the public in Hong Kong. Majority (60.2%) of the population reported having a regular primary care doctor and one third (36.5%) had a regular family doctor. The emergency service was used by a significant 7.3% of the population for primary care during the last episode of illness, and at even a higher prevalence (9.6%) among people without a regular primary care doctor. The findings supported the adoption of a family-doctor led primary care service in Hong Kong and possibly other Asian countries. The challenge is how to help every citizen find a regular family doctor and to enable more primary care doctors to become family doctors. The public should have access to more information on primary care practitioners so that they can choose one who can serve as their family doctors. Better manpower planning with more postgraduate family medicine training opportunities is urgently needed to increase the supply of family doctors.

## Additional file

Additional file 1:
**1: Notes on Independent Variables in the Regression Analyses.** **2:** Full details of regression coefficients of all independent variables in the logistic, Poisson and Negative binomial regression analyses.

## Abbreviations

RFD: Regular family doctor group
ORD: Other regular doctor group
NRD: No regular doctor group
US: Unites States
UK: United Kingdom
