# Supplementary material for: Having a family doctor was associated with lower utilization of hospital-based health services
Source: BMC Health Serv Res. 2015 Jan 28;15:42. doi: 10.1186/s12913-015-0705-7 (PMC4312460; doi:10.1186/s12913-015-0705-7)
Supplement: Additional file 1: — 1: Notes on Independent Variables in the Regression Analyses. 2: Full details of regression coefficients of all independent variables in the logistic, Poisson and Negative binomial regression analyses. [file 12913_2015_705_MOESM1_ESM.docx]

Appendix 1 – Notes on Independent Variables in the Regression Analyses

| **Variable** | **Measurement** | **Notes** |
| --- | --- | --- |
|  |  |  |
| **Type of Primary Care Doctor groups** | | |
| Doctor choice groups | Four categories: ‘regular not family doctor’, ‘regular family doctor’, ‘no regular doctor’ and ‘not sure’ | (a) ‘no regular doctor’ is the  reference category  (b) ‘regular not family  doctor’ is the reference  category |
|  |  |  |
| **Socio-demographics** | | |
| Age | Continuous variable |  |
| Sex | Two categories: ‘female’ and ‘male’ | ‘male’ is the reference category |
| Occupation | Originally ten categories recorded as seven: ‘managerial/ administrative/ professional/ employer’, ‘white-collar worker’, ‘blue-collar worker/ service and sales worker’, ‘student’, ‘home-maker’, ‘retired/ unemployed’ and ‘others’ | ‘blue-collar worker/ service and sales worker’ is the reference category |
| Marital status | Originally five categories recorded as three: ‘single/ divorced/ widower’, ‘married’ and ‘refuse to answer’ | ‘single/ divorced/ widower’ is the reference category |
| Household monthly income | Seven categories: ‘<$5,000’, ‘$5,000-9,999’, ‘$10,000-19,999’, ‘$20,000-29,999’, ‘$30,000-39,999’, ‘>=$40,000’ and ‘refuse to answer’ | ‘<$5,000’ is the reference category |
| Education level | Originally five categories recorded as four: ‘nil/ primary’, ‘secondary’, ‘tertiary’ and ‘refuse to answer’ | ‘nil/ primary’ is the reference category |
| District | Nineteen categories: ‘Central & Western’, ‘Wan Chai’, ‘Eastern’, ‘Southern’, ‘Yau Tsim Mong’, ‘Sham Shui Po’, ‘ Kowloon City’, ‘Wong Tai Sin’, ‘Kwun Tong’, ‘Kwai Tsing’, ‘Tsuen Wan’, ‘Tuen Mun’, ‘Yuen Long’, ‘North’, ‘Tai Po’, ‘Sha Tin’, ‘Sai Kung’, ‘Islands’ and ‘not sure’ | ‘Islands’ is the reference category |
|  |  |  |
| **Health status** | | |
| General health | Five categories: ‘excellent’, ‘very good’, ‘good’, ‘fair’ and ‘poor’ | ‘poor’ is the reference category |
|  |  |  |
| **Chronic Morbidity** | | |
| Chronic Disease | Two categories: ‘no/ not sure’ and ‘yes’ | ‘yes’ is the reference category |
| Long-term medication | Two categories: ‘no/ not sure’ and ‘yes’ | ‘yes’ is the reference category |
| **Lifestyle** | | |
| Smoking | Originally six categories recorded as two: ‘no/ don’t know’ and ‘yes’ | ‘yes’ is the reference category |
| Drinking | Originally six categories recorded as two: ‘no/ don’t know’ and ‘yes’ | ‘yes’ is the reference category |
| Regular exercise | Originally five categories recorded as two: ‘no/ don’t know’ and ‘yes’ | ‘no/ don’t know’ is the reference category |
|  |  |  |
| **Seasonality** |  |  |
| seasonality | Two categories: ‘phase 1’ and ‘phase 2’ | ‘phase 2’ is the reference category |
|  |  |  |

Appendix 2 – Full details of regression coefficients of all independent variables in the logistic, Poisson and Negative binomial regression analyses.

| Effects of primary care doctor choice on any primary care doctor consultation during the last episode of illness by logistic regression | | | | | | | | | | | | | | |
| --- | --- | --- | --- | --- | --- | --- | --- | --- | --- | --- | --- | --- | --- | --- |
|  | | | | P-value | | | Odds Ratio | | | 95%CI | | | | |
| Drinking | | | | 0.006 | | | 1.264 | | | 1.068 | | | 1.497 | |
| Regular exercise | | | | 0.016 | | | 1.229 | | | 1.039 | | | 1.454 | |
| Occupation (Others) | | | | 0.001 | | |  | | |  | | |  | |
| Managerial/Administrative/Professional/Employer | | | | 0.564 | | | 1.090 | | | 0.813 | | | 1.461 | |
| White-collar worker | | | | 0.017 | | | 1.406 | | | 1.063 | | | 1.858 | |
| Blue-collar worker/Service and sales worker | | | | 0.342 | | | 0.870 | | | 0.652 | | | 1.160 | |
| Student | | | | 0.022 | | | 0.728 | | | 0.555 | | | 0.954 | |
| Home-maker | | | | 0.962 | | | 1.007 | | | 0.770 | | | 1.315 | |
| Retired/Unemployed | | | | 0.392 | | | 0.744 | | | 0.378 | | | 1.464 | |
| Marital status (Refuse to answer) | | | | 0.012 | | |  | | |  | | |  | |
| Single/Divorced/Widower | | | | 0.003 | | | 1.339 | | | 1.101 | | | 1.628 | |
| Married | | | | 0.883 | | | 0.945 | | | 0.442 | | | 2.020 | |
| Doctor group (NRD) | | | | <0.001 | | |  | | |  | | |  | |
| Not sure | | | | 0.003 | | | 2.132 | | | 1.288 | | | 3.529 | |
| ORD | | | | <0.001 | | | 1.853 | | | 1.509 | | | 2.275 | |
| RFD | | | | <0.001 | | | 2.486 | | | 2.053 | | | 3.010 | |
| Intercept | | | | 0.697 | | | 1.052 | | |  | | |  | |
|  | | | |  | | |  | | |  | | |  | |
| Effects of primary care doctor choice on emergency service visit during the last episode of illness by logistic regression | | | | | | | | | | | | | |  |
|  | | | P-value | | | Odds Ratio | | | 95%CI | | | | |  |
| Age | | | 0.008 | | | 1.013 | | | 1.003 | | | 1.023 | |  |
| General health (Poor) | | | 0.015 | | |  | | |  | | |  | |  |
| Excellent | | | 0.916 | | | 0.959 | | | 0.444 | | | 2.070 | |  |
| Very good | | | 0.057 | | | 0.562 | | | 0.310 | | | 1.016 | |  |
| Good | | | 0.002 | | | 0.410 | | | 0.233 | | | 0.721 | |  |
| Fair | | | 0.029 | | | 0.596 | | | 0.374 | | | 0.947 | |  |
| Long-term medication | | | 0.020 | | | 0.654 | | | 0.458 | | | 0.935 | |  |
| Household monthly income (Refuse to answer) | | | 0.005 | | |  | | |  | | |  | |  |
| <$5,000 | | | 0.400 | | | 0.809 | | | 0.494 | | | 1.324 | |  |
| $5,000-10,000 | | | 0.051 | | | 0.615 | | | 0.377 | | | 1.003 | |  |
| $10,000-20,000 | | | 0.209 | | | 0.705 | | | 0.409 | | | 1.216 | |  |
| $20,000-30,000 | | | 0.011 | | | 0.387 | | | 0.186 | | | 0.807 | |  |
| $30,000-40,000 | | | <0.001 | | | 0.315 | | | 0.162 | | | 0.613 | |  |
| >$40,000 | | | 0.007 | | | 0.449 | | | 0.252 | | | 0.803 | |  |
| District (not sure) | | | 0.023 | | |  | | |  | | |  | |  |
| Central & Western | | | 0.251 | | | 2.740 | | | 0.490 | | | 15.319 | |  |
| Wan Chai | | | 0.830 | | | 1.205 | | | 0.219 | | | 6.640 | |  |
| Eastern | | | 0.557 | | | 0.478 | | | 0.041 | | | 5.622 | |  |
| Southern | | | 0.680 | | | 1.380 | | | 0.299 | | | 6.374 | |  |
| Yau Tsim Mong | | | 0.563 | | | 1.611 | | | 0.320 | | | 8.100 | |  |
| Sham Shui Po | | | 0.791 | | | 1.247 | | | 0.244 | | | 6.375 | |  |
| Kowloon City | | | 0.196 | | | 2.735 | | | 0.596 | | | 12.562 | |  |
| Wong Tai Sin | | | 0.395 | | | 1.960 | | | 0.416 | | | 9.246 | |  |
| Kwun Tong | | | 0.721 | | | 0.743 | | | 0.146 | | | 3.790 | |  |
| Kwai Tsing | | | 0.272 | | | 2.307 | | | 0.519 | | | 10.262 | |  |
| Tsuen Wan | | | 0.861 | | | 1.151 | | | 0.238 | | | 5.561 | |  |
| Tuen Mun | | | 0.606 | | | 0.629 | | | 0.108 | | | 3.653 | |  |
| Yuen Long | | | 0.889 | | | 1.118 | | | 0.233 | | | 5.360 | |  |
| North | | | 0.191 | | | 2.733 | | | 0.606 | | | 12.325 | |  |
| Tai Po | | | 0.453 | | | 1.832 | | | 0.377 | | | 8.897 | |  |
| Sha Tin | | | 0.411 | | | 1.957 | | | 0.394 | | | 9.709 | |  |
| sai Kung | | | 0.390 | | | 1.941 | | | 0.428 | | | 8.799 | |  |
| Islands | | | 0.142 | | | 3.099 | | | 0.686 | | | 14.001 | |  |
| Doctor group (NRD) | | | <0.001 | | |  | | |  | | |  | |  |
| Not sure | | | 0.287 | | | 1.440 | | | 0.736 | | | 2.819 | |  |
| ORD | | | 0.148 | | | 0.768 | | | 0.536 | | | 1.098 | |  |
| RFD | | | <0.001 | | | 0.479 | | | 0.330 | | | 0.695 | |  |
| Intercept | | | 0.012 | | | 0.124 | | |  | | |  | |  |
|  | | |  | | |  | | |  | | |  | |  |
|  | | |  | | |  | | |  | | |  | |  |
| Effects of primary care doctor choice on hospital admission during the last episode of illness by logistic regression | | | | | | | | | | |  |  |  |  |
|  | P-value | Odds Ratio | | | 95%CI | | | | | |  |  |  |  |
| Age | 0.002 | 1.021 | | | 1.007 | | | 1.035 | | |  |  |  |  |
| Long-term medication | 0.006 | 0.514 | | | 0.319 | | | 0.828 | | |  |  |  |  |
| Doctor group (NRD) | 0.032 |  | | |  | | |  | | |  |  |  |  |
| Not sure | 0.778 | 1.149 | | | 0.439 | | | 3.009 | | |  |  |  |  |
| ORD | 0.652 | 0.891 | | | 0.540 | | | 1.470 | | |  |  |  |  |
| RFD | 0.005 | 0.458 | | | 0.267 | | | 0.788 | | |  |  |  |  |
| Intercept | <0.001 | 0.025 | | |  | | |  | | |  |  |  |  |
|  |  |  | | |  | | |  | | |  |  |  |  |

| Effects of primary care doctor choice on Monthly consultation rate by Negative Binomial regression | | | | |
| --- | --- | --- | --- | --- |
|  | Coefficient | 95%CI | | P-value |
| Intercept | -2.870 | -3.931 | -1.809 | <0.001 |
| Male | -0.406 | -0.540 | -0.272 | <0.001 |
|  |  |  |  |  |
| General health (Poor) | |  |  | <0.001 |
| Excellent | 1.413 | 0.991 | 1.836 | <0.001 |
| Very good | 0.850 | 0.471 | 1.229 | <0.001 |
| Good | 0.419 | 0.033 | 0.805 | 0.034 |
| Fair | 0.104 | -0.297 | 0.506 | 0.611 |
|  |  |  |  |  |
| Chronic disease | 0.352 | 0.221 | 0.484 | <0.001 |
|  |  |  |  |  |
| Drinking | -0.150 | -0.278 | -0.022 | 0.021 |
|  |  |  |  |  |
| Occupation (Managerial/Administrative/Professional/Employer) | | | | 0.014 |
| Blue-collar worker/Service and sales worker | 0.198 | -0.027 | 0.424 | 0.085 |
| Others | 0.003 | -0.546 | 0.551 | 0.993 |
| Retired/Unemployed | -0.056 | -0.296 | 0.184 | 0.647 |
| Home-maker | -0.145 | -0.389 | 0.099 | 0.244 |
| Student | 0.059 | -0.174 | 0.292 | 0.618 |
| White-collar worker | 0.170 | -0.050 | 0.391 | 0.129 |
|  |  |  |  |  |
| Education (Refuse to answer) | | |  | 0.001 |
| Nil/Primary | 0.263 | -0.499 | 1.025 | 0.498 |
| Secondary | 0.236 | -0.515 | 0.988 | 0.538 |
| Tertiary | 0.529 | -0.227 | 1.285 | 0.170 |
|  |  |  |  |  |
| District (not sure) | |  |  | 0.008 |
| Central & Western | 0.883 | 0.093 | 1.673 | 0.028 |
| Islands | 1.196 | 0.333 | 2.059 | 0.007 |
| sai Kung | 1.165 | 0.408 | 1.922 | 0.003 |
| Sha Tin | 1.092 | 0.341 | 1.843 | 0.004 |
| Tai Po | 0.902 | 0.113 | 1.691 | 0.025 |
| North | 1.194 | 0.421 | 1.968 | 0.002 |
| Yuen Long | 0.997 | 0.240 | 1.753 | 0.010 |
| Tuen Mun | 1.246 | 0.493 | 1.999 | 0.001 |
| Tsuen Wan | 1.231 | 0.471 | 1.991 | 0.001 |
| Kwai Tsing | 1.005 | 0.247 | 1.763 | 0.009 |
| Kwun Tong | 1.259 | 0.512 | 2.005 | <0.001 |
| Wong Tai Sin | 0.756 | -0.011 | 1.523 | 0.053 |
| Kowloon City | 1.065 | 0.300 | 1.831 | 0.006 |
| Sham Shui Po | 1.185 | 0.415 | 1.955 | 0.003 |
| Yau Tsim Mong | 0.839 | 0.059 | 1.619 | 0.035 |
| Southern | 0.919 | 0.133 | 1.706 | 0.022 |
| Eastern | 1.043 | 0.293 | 1.792 | 0.006 |
| Wan Chai | 0.586 | -0.328 | 1.500 | 0.209 |
|  |  |  |  |  |
| Doctor group (Not sure) | |  |  | <0.001 |
| NRD | -0.007 | -0.393 | 0.378 | 0.970 |
| RFD | 0.466 | 0.083 | 0.848 | 0.017 |
| ORD | 0.420 | 0.032 | 0.807 | 0.034 |
|  |  |  |  |  |
| Seasonality | 0.329 | 0.212 | 0.447 | <0.001 |
|  |  |  |  |  |

| Effects of primary care doctor choice on Monthly emergency service visit rate by Poisson regression | | | | |
| --- | --- | --- | --- | --- |
|  | Coefficient | 95%CI | | P-value |
| Intercept | -29.863 | -32.201 | -27.525 | <0.001 |
| Male | 0.581 | 0.229 | 0.933 | 0.001 |
|  |  |  |  |  |
| Age | -0.017 | -0.031 | -0.002 | 0.029 |
| General health (Poor) | |  |  | <0.001 |
| Excellent | 2.421 | 1.214 | 3.628 | <0.001 |
| Very good | 1.261 | 0.095 | 2.427 | 0.034 |
| Good | -0.270 | -1.543 | 1.002 | 0.677 |
| Fair | 0.458 | -0.785 | 1.701 | 0.470 |
|  |  |  |  |  |
| Long-term medication | 0.474 | 0.087 | 0.861 | 0.016 |
|  |  |  |  |  |
| Occupation (Managerial/Administrative/Professional/Employer) | | | | 0.003 |
| Blue-collar worker/Service and sales worker | 1.888 | 0.812 | 2.965 | <0.001 |
| Others | 0.400 | -1.829 | 2.630 | 0.725 |
| Retired/Unemployed | 2.038 | 0.926 | 3.151 | <0.001 |
| Home-maker | 1.849 | 0.733 | 2.965 | 0.001 |
| Student | 1.757 | 0.619 | 2.895 | 0.002 |
| White-collar worker | 1.114 | -0.042 | 2.271 | 0.059 |
|  |  |  |  |  |
| Household monthly income (Refuse to answer) | | | | <0.001 |
| >$40,000 | 0.760 | -0.066 | 1.585 | 0.071 |
| $30,000-40,000 | 1.595 | 0.860 | 2.331 | <0.001 |
| $20,000-30,000 | 0.263 | -0.511 | 1.037 | 0.506 |
| $10,000-20,000 | 0.680 | -0.116 | 1.476 | 0.094 |
| $5,000-10,000 | -0.113 | -1.167 | 0.942 | 0.834 |
| <$5,000 | 0.865 | 0.044 | 1.685 | 0.039 |
|  |  |  |  |  |
| Education (Refuse to answer) | | |  | 0.013 |
| Nil/Primary | 23.390 | 22.801 | 23.979 | <0.001 |
| Secondary/Tertiary | 24.043 | 23.600 | 24.487 | <0.001 |
|  |  |  |  |  |
|  |  |  |  |  |
| District (not sure) | |  |  | <0.001 |
| Central & Western | -0.910 | -2.905 | 1.086 | 0.372 |
| Islands | 0.003 | -1.991 | 1.996 | 0.998 |
| sai Kung | 0.768 | -0.722 | 2.258 | 0.312 |
| Sha Tin | 0.130 | -1.377 | 1.637 | 0.866 |
| Tai Po | 0.224 | -1.382 | 1.830 | 0.784 |
| North | 0.221 | -1.347 | 1.789 | 0.783 |
| Yuen Long | 0.736 | -0.741 | 2.214 | 0.329 |
| Tuen Mun | -0.019 | -1.544 | 1.505 | 0.980 |
| Tsuen Wan | -1.467 | -3.449 | 0.514 | 0.147 |
| Kwai Tsing | -1.007 | -2.735 | 0.721 | 0.253 |
| Kwun Tong | -0.190 | -1.697 | 1.317 | 0.805 |
| Wong Tai Sin | -0.512 | -2.117 | 1.093 | 0.532 |
| Kowloon City | -0.479 | -2.145 | 1.187 | 0.573 |
| Sham Shui Po | -0.247 | -1.857 | 1.363 | 0.764 |
| Yau Tsim Mong | 0.108 | -1.495 | 1.711 | 0.895 |
| Southern | -0.748 | -2.734 | 1.237 | 0.460 |
| Eastern | 0.482 | -1.015 | 1.979 | 0.528 |
| Wan Chai | 0.866 | -0.810 | 2.542 | 0.311 |
|  |  |  |  |  |
| Doctor group (Not sure) | |  |  | 0.158 |
| NRD | -0.748 | -1.571 | 0.076 | 0.075 |
| RFD | -0.494 | -1.325 | 0.336 | 0.243 |
| ORD | -0.395 | -1.223 | 0.432 | 0.349 |
| Not sure |  |  |  |  |
| Seasonality | 0.520 | 0.196 | 0.844 | 0.002 |
|  |  |  |  |  |

| Effects of primary care doctor choice on Monthly hospital admission rate by Poisson regression | | | | |
| --- | --- | --- | --- | --- |
|  | Coefficient | 95%CI | | P-value |
| Intercept | -3.687 | -4.847 | -2.528 | <0.001 |
| Seasonality | 0.710 | 0.093 | 1.327 | 0.024 |
|  |  |  |  |  |
| Doctor group (Not sure) | |  |  | 0.360 |
| NRD | -1.043 | -2.290 | 0.205 | 0.101 |
| RFD | -1.090 | -2.342 | 0.163 | 0.088 |
| ORD | -0.836 | -2.110 | 0.437 | 0.198 |
|  |  |  |  |  |
|  |  |  |  |  |
